# Genomic insights into *Penicillium chrysogenum* adaptation to subseafloor sedimentary environments

**DOI:** 10.1186/s12864-023-09921-1

**Published:** 2024-01-02

**Authors:** Xuan Liu, Xinran Wang, Fan Zhou, Yarong Xue, Changhong Liu

**Affiliations:** State Key Laboratory of Pharmaceutical Biotechnology, Nanjing University, Nanjing, 210023 China

**Keywords:** *Penicillium chrysogenum*, Subseafloor sediment, Secondary metabolites, Environmental adaptation, Fungi

## Abstract

**Background:**

*Penicillium chrysogenum* is a filamentous fungal species with diverse habitats, yet little is known about its genetics in adapting to extreme subseafloor sedimental environments.

**Results:**

Here, we report the discovery of *P. chrysogenum* strain 28R-6-F01, isolated from deep coal-bearing sediments 2306 m beneath the seafloor. This strain possesses exceptional characteristics, including the ability to thrive in extreme conditions such as high temperature (45 °C), high pressure (35 Mpa), and anaerobic environments, and exhibits broad-spectrum antimicrobial activity, producing the antibiotic penicillin at a concentration of 358 μg/mL. Genome sequencing and assembly revealed a genome size of 33.19 Mb with a GC content of 48.84%, containing 6959 coding genes. Comparative analysis with eight terrestrial strains identified 88 unique genes primarily associated with penicillin and aflatoxins biosynthesis, carbohydrate degradation, viral resistance, and three secondary metabolism gene clusters. Furthermore, significant expansions in gene families related to DNA repair were observed, likely linked to the strain’s adaptation to its environmental niche.

**Conclusions:**

Our findings provide insights into the genomic and biological characteristics of *P. chrysogenum* adaptation to extreme anaerobic subseafloor sedimentary environments, such as high temperature and pressure.

**Supplementary Information:**

The online version contains supplementary material available at 10.1186/s12864-023-09921-1.

## Background

Deep subseafloor sediment provides a unique microbial habitat that supports a significant portion of the global microbial population and organic carbon reserves [1, 2]. Microorganisms residing in this environment face numerous challenges, including high pressure, limited nutrient availability, low oxygen levels, and extreme temperature fluctuations [3–5]. While extensive research has been conducted on prokaryotes, recent studies have highlighted the importance of fungi in the subseafloor biosphere, with some species found at depths of up to approximately 2.5 km below the seafloor [6, 7]. Both culture-dependent and culture-independent methods have confirmed the metabolic activity of fungi in deep subseafloor sediments [8–10].

Studies have revealed distinctive evolutionary processes, physiological characteristics, and energy metabolism features in subseafloor fungi that enable their survival and growth in anaerobic and high pressure environments. For example, the basidiomycete fungus *Schizophyllum commune*, isolated from coal-bearing sediments approximately 2.0 km beneath the seafloor, exhibits lower nucleotide diversity, substitution rate, and homologous recombination compared to terrestrial strains. This fungus also shows significant expansion of genes encoding FunK1 protein kinase, NmrA family, transposons, and DNA repair [11]. Additionally, *S. commune* possesses special energy generation processes through ethanol fermentation and amino acid metabolism, as well as an increase in mitochondrial number [12, 13]. It also has complex nitrogen conversion mechanisms under anaerobic conditions and the ability to degrade difficult-to-degrade macromolecules such as lignite, resulting in the production of methane and extracellular polysaccharides [14–16]. Simonato et al. reported that the yeast *Saccharomyces cerevisiae* can modify its membrane composition to tolerate high hydrostatic pressure [17]. Understanding the biological and genetic mechanisms underlying fungal adaptation to subseafloor sedimentary environments can provide valuable insights into their survival in extreme conditions.

*Penicillium chrysogenum*, a filamentous fungus known for its production of the antibiotic penicillin, has been found to inhabit diverse terrestrial and aquatic habitats, including subseafloor sedimentary environments [18, 19]. It has been demonstrated that persistent *P. chrysogenum* strains in subseafloor sediment exhibit distinct metabolic activities, such as formaldehyde metabolism and resistance to heavy metal stress [20, 21]. Several subseafloor strains of *P. chrysogenum* have been discovered to produce novel secondary metabolites and display antimicrobial activities [22–24]. However, the genomic features of these *P. chrysogenum* strains involved in secondary metabolism and environmental adaptation remain unknown.

In this study, we present a novel strain 28R-6-F01 of *P. chrysogenum*, which was isolated from anaerobic coal-bearing sediments 2306 m beneath the seafloor at high temperatures (45 °C) and high pressures (35 Mpa) during the Integrated Ocean Drilling Program (IODP) Expedition 337. The aim of this study is to uncover the key genetic features that may contribute to the survival, growth, and adaptation of strain 28R-6-F01 in extreme subseafloor environments, achieved by sequencing the genome of this strain and conducting comparative analysis with the genomes of corresponding terrestrial strains.

## Results and discussion

### Identification of strain 28R-6-F01

**Fig. 1:**
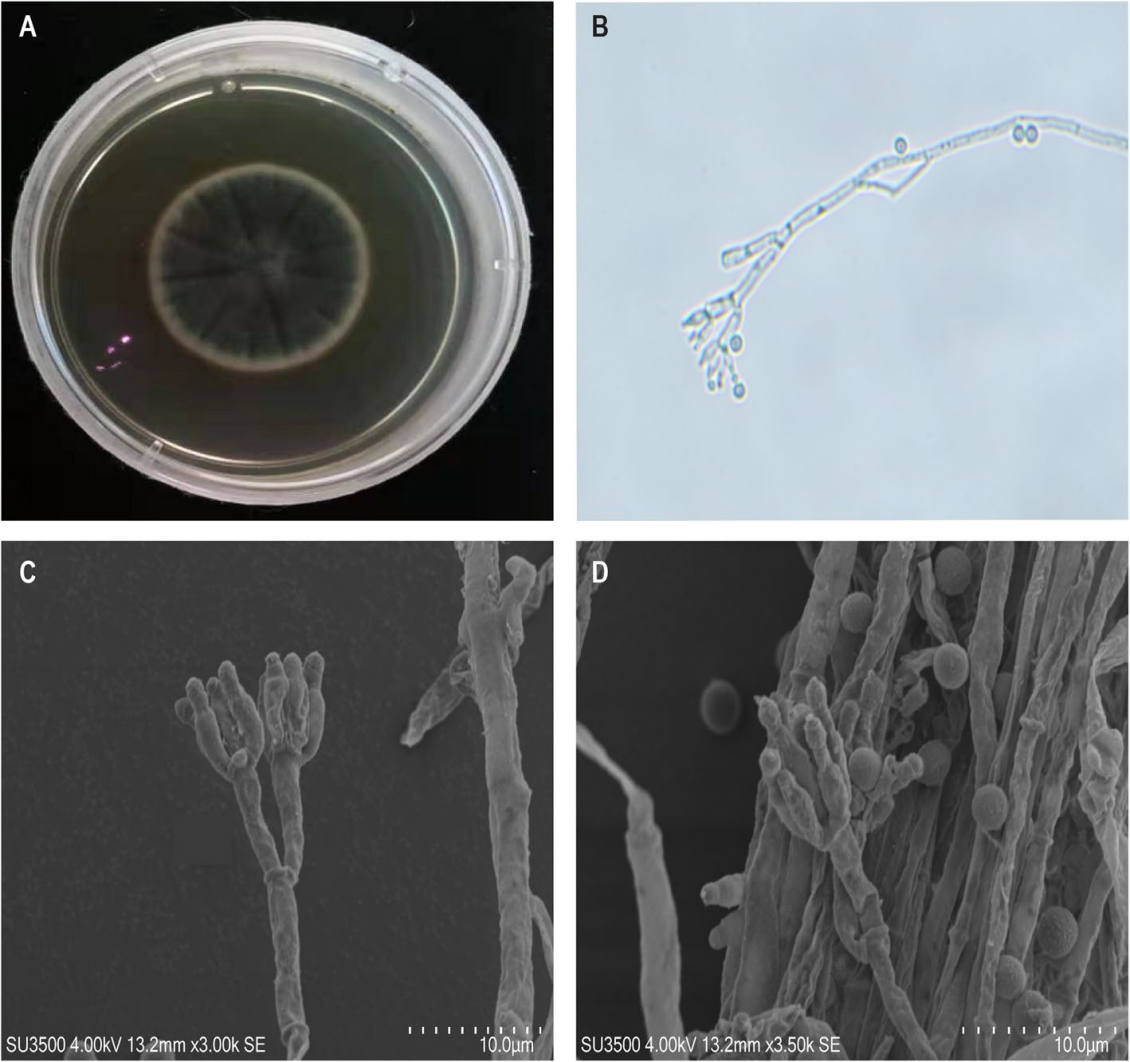
Colony features of *P. chrysogenum* 28R-6-F01. **A**, colony morphology. **B**, hyphae morphology under optical microscope. **C** and **D**, microscopic features of the colonies, hyphae and spores under scanning electron microscope

Strain 28R-6-F01 exhibited green and dense furry surface with white edge and produced yellow pigmented colonies when cultured on PDA plates at 30 °C for 5 days (Fig. 1A). Microscopic observation showed transparent, tubular, branched hyphae with septa (Fig. 1B), producing brush-like clusters of branching conidiophores, intermediate branches, and spherical spores (20–30 μm diameter) (Fig. 1C, D). These characteristics are consistent with *P. chrysogenum* strain Y5 morphology [25]. Additionally, the homology comparison of the ITS gene sequence confirmed that strain 28R-6-F01 is indeed *P. chrysogenum* and shares complete identity with the B11 strain.

### Growth capacity

**Fig. 2:**
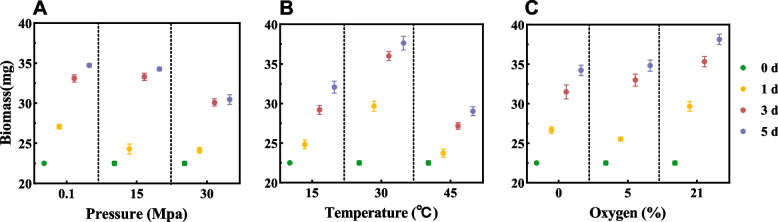
Growth of *P. chrysogenum* 28R-6-F01 in different conditions. **A**-**C**, represent the biomass of pressure, temperature, and oxygen, respectively. Error bars indicate standard deviations

The study cultured *P. chrysogenum* 28R-6-F01 under simulated in situ conditions (45 °C, 35 Mpa, 0% oxygen). The fungus showed gradual increase in biomass over time, indicating its ability to grow under similar conditions (Fig. 2). However, compared to milder conditions, the growth of this strain was slower and resulted in lower biomass. Previous studies have shown variations in *P. chrysogenum* strains’ tolerance to high temperature and pressure. For example, the DY-F2 strain from East Pacific sediment 1674 m beneath the seafloor can grow at 45 °C [20], similar to our strain. In contrast, strains from South China Sea sediment 15 m below the seafloor cannot grow above 45 °C [26]. The A57 strain from 3500 m Mariana Trench sediment can grow under 20 Mpa pressure [27], while our strain grew under 35 Mpa conditions. These differences may be due to genetic diversity and adaptability of *P. chrysogenum* strains.

### Antimicrobial activities and penicillin production

**Fig. 3:**
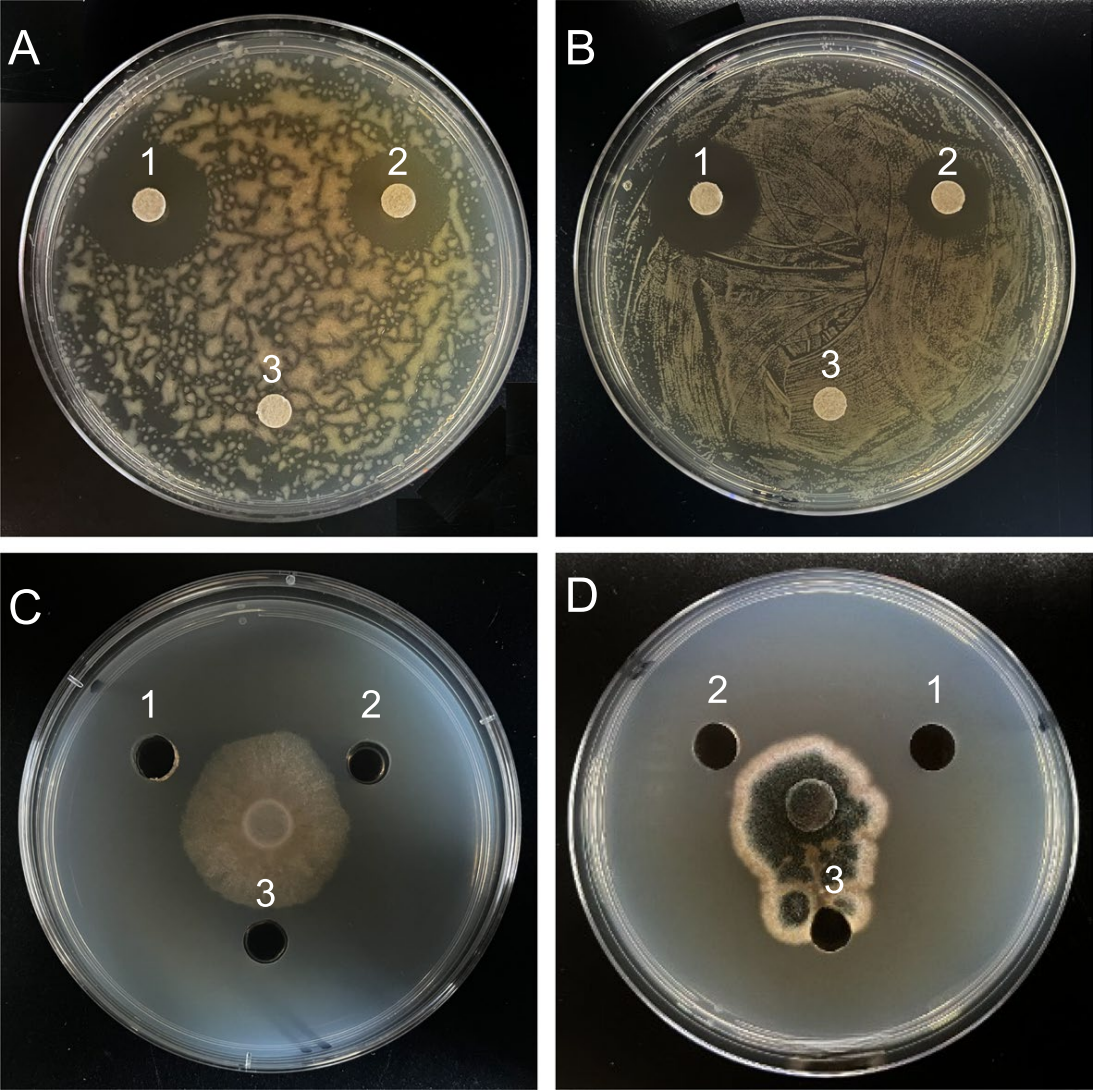
Antimicrobial activity of *P. chrysogenum* 28R-6-F01. **A**-**D**, the inhibitory activity of the fermentation broths on *B. subtilis* and *E. coli*, *P. capsici* and *A. sydowii*, respectively. 1–3 represent positive control, tested microorganisms, and sterile water, respectively

The antibacterial and antifungal activities of *P. chrysogenum* strain 28R-6-F01 culture filtrate was assessed using the paper disc diffusion assay and the agar diffusion assay, respectively. The filtrate showed inhibition against both Gram-positive (*Bacillus subtilis*) and Gram-negative bacteria (*Escherichia coli*) (Fig. 3A, B). It also exhibited inhibitory activity against *Phytophthora capsici* and *Aspergillus sydowii* fungi (Fig. 3C, D). After 24 h of incubation at 30 °C, the inhibition zone diameters were measured as 20 mm for *B. subtilis* and 16 mm for *E. coli*. For *A. sydowii* and *P. capsici*, the inhibition rates were 18.23 and 20.57%, respectively. These results indicate that *P. chrysogenum* strain 28R-6-F01, isolated from deep subseafloor sediment, has a broader antimicrobial spectrum compared to the land-derived strain IFL1 [28].

Furthermore, the production of penicillin by strain 28R-6-F01 was investigated due to the industrial importance of *P. chrysogenum* in penicillin production [29]. After 72 h of incubation at 30 °C, the strain produced 358 μg/mL of penicillin (Additional file 1: Fig. S1, S2). This production level exceeds that of the terrestrial strain Wisconsin54–1255, which produces 180 μg/mL of penicillin [30]. These findings suggest that the deep subseafloor sediment-derived *P. chrysogenum* strain 28R-6-F01 has the potential for large-scale industrial production of penicillin. Additionally, it indicates that this strain may possess unique ecological adaptations that provide a competitive advantage in its natural habitat.

### Genome assembly and annotation

**Fig. 4:**
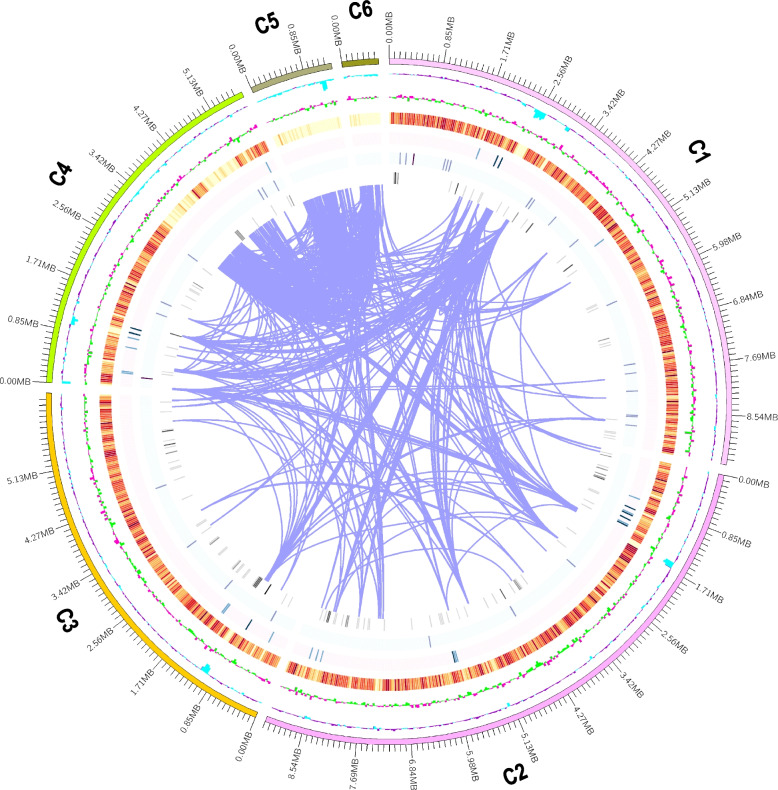
The circos diagram of *P. chrysogenum* 28R-6-F01 genome. The outermost layer is the chromosome and its size. The second layer is GC content, the blue part indicates that the GC content in this area is lower than the whole genome average GC content, and the purple part indicates that the GC content in this area is higher than the whole genome average GC content. The third layer is GC skew (G-C/G + C), the green part indicates that the G content in this area is lower than the C content, and the pink part indicates that the G content in this area is higher than the C content. The fourth to seventh layers are gene density for CDS, rRNA, snRNA, and tRNA, respectively. The eighth layer is the chromosome duplication

The strain 28R-6-F01 of *P. chrysogenum* was sequenced using Nanopore technology. After filtering out low-quality reads, over 6.54 Gb (~ 200 ×) of high-quality reads were assembled into a genome size of 33.19 Mb, consisting of 10 contigs. The contig N50 was 9.14 Mb, and the GC content was 48.84% (Fig. 4, Table 1). The assembly quality of the 28R-6-F01 genome was comparable to the terrestrial strain P2niaD18 but better than other terrestrial strains like Wisconsin54–1255, KF-25, and NCPC10086 (Additional file 2: Table S1). The genome size and GC content of this strain were similar to other environmental strains of *P. chrysogenum* (Table 1, Additional file 2: Table S2). This suggests that horizontal gene transfer or hybridization had limited impact on the strain’s evolution in deep subseafloor sediments over millions of years [11, 31]. Additionally, the mitochondrial genome of 28R-6-F01 was assembled, which had a size of 26,185 bp and contained 17 genes, including 7 NADH dehydrogenase, 3 cytochrome oxidase, 1 cytochrome b, 3 ribosomal protein, and 3 ATP synthase genes. The size and gene number of the mitochondrial genome were similar to strains P2niaD18 and Wisconsin54–1255 [32, 33].

**Table 1:** Genome assembly and annotation summary of *P. chrysogenum* strains

Strains	Size (Mb)	Scaffold	N50	GC (%)	Gene number	Average gene length (bp)	Average exon length (bp)	Average intron length (bp)
28R-6-F01	33.19	10	9.14	48.84	6959	1527	441	93
IBT17219	33.64	12	4.19	48.95	12,458	1500	436	78
IBT19737	35.18	15	4.88	48.8	12,547	1508	458	81
IBT3361	33.93	12	4.64	48.84	12,251	1515	457	80
IBT35668	32.41	5	9.49	48.92	11,980	1520	455	79
P2niaD18	32.53	5	10.46	48.95	11,460	1861	580	74
Wisconsin54–1255	32.22	49	3.89	48.96	12,493	1509	360	89
Pench1	31.34	27	5.34	48.78	11,396	1590	451	89
ITEM4680	32.80	258	1.57	48.87	12,009	1928	452	87

The genome of *P. chrysogenum* 28R-6-F01 was annotated using ab initio prediction and homology-based approaches, resulting in the identification of 6959 protein-coding genes. More than 98.12% of these genes were successfully annotated for their functions in various databases such as Gene Ontology (GO), Kyoto Encyclopedia of Genes and Genomes (KEGG), Clusters of Orthologous Groups (COG), Protein Families Database (Pfam), Swiss-Prot protein sequence database (SwissProt), and Non-redundant protein sequence database (NR) (Additional file 3: Table S1). This indicates the accuracy of the gene predictions. The average length of genes, exons, and introns were determined to be 1527 bp, 441 bp, and 93 bp, respectively (Table 1). A total of 2577 repetitive elements, accounting for 1.91% of the genome, were identified in strain 28R-6-F01 (Additional file 3: Table S2). This is lower than strain KF-25 but higher than strain Wisconsin 54–1255 [32, 34]. Additionally, the genome contains 186 tRNA, 42 rRNA, and 34 snRNA genes, which are non-coding RNA genes (Additional file 3: Table S3).

### Genome comparison of *P. Chrysogenum* strains

The genomic analysis revealed that strain 28R-6-F01 exhibited 88 unique genes associated with secondary metabolism, DNA repair, and carbohydrate hydrolysis, compared to eight corresponding terrestrial strains (Additional file 2: Table S3). Additionally, seven gene families were expanded and 115 were contracted in the strain (Additional file 2: Table S4). Among the expanded gene families, six were identified as Polyketide synthase (PKS), S-(hydroxymethyl) glutathione dehydrogenase, UvrABC system protein A, Integrase, DASH complex subunit Dad2, and disulfide oxidoreductase, while one remained unknown.

#### Secondary metabolites

**Fig. 5:**
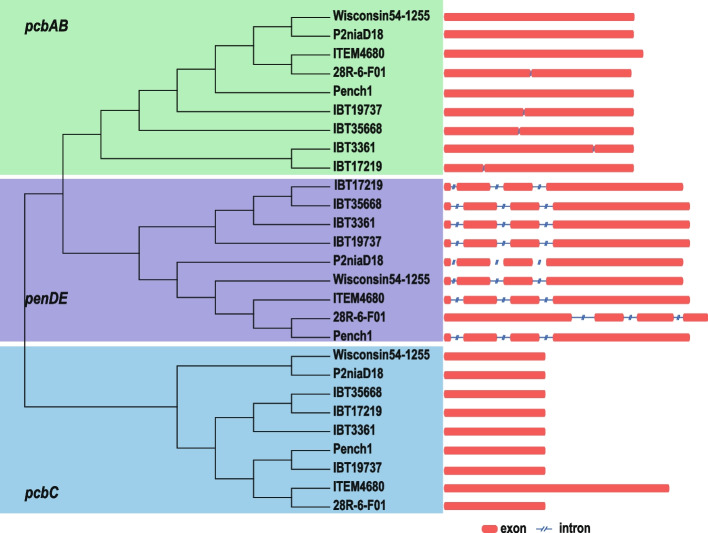
Phylogenetic relationships and gene structure of penicillin biosynthetic genes in different *P. chrysogenum* strains

*P. chrysogenum* is known for its significant metabolic feature, which is the production of penicillin. This process involves a penicillin biosynthetic gene cluster (*pcbAB*, *pcbC*, and *penDE*) and three transport genes (*penM*, *paaT*, and *penV*) [35]. Although these genes are present in the genome of the 28R-6-F01 strain (Additional file 1: Fig. S3, S4), their gene structure sequence of *penDE* and the positions of *penM* and *penV* on the chromosome differ from those in eight tested terrestrial strains (Fig. 5, Additional file 2: Table S5). This suggests that *P. chrysogenum*, which has settled in deep subseafloor sediment for millions of years, may have different penicillin metabolism activities compared to terrestrial strains. Our preliminary research results show that under the same culture conditions, the 28R-6-F01 strain produces almost twice as much penicillin as the terrestrial strain Wisconsin54–1255 [32]. Further research is necessary to understand how the differences in gene structure sequence of *penDE* and positions of *penM* and *penV* on the chromosome affect penicillin synthesis in *P. chrysogenum*.

Furthermore, we have identified 45 gene clusters associated with secondary metabolism in the genome of *P. chrysogenum* 28R-6-F01. These clusters encompass 12 non-ribosomal peptides (NRPS), 11 NRPS-like, 16 polyketides, three terpenes, one indole, one betalactone, and one NRP-metallophore (Additional file 3: Table S4). Notably, within these clusters, seven encode mycotoxins and antibiotics, such as aspercryptins, nidulanin A, patulin, penicillin, metachelin, trans-resorcylide, and azasperpyranone (Additional file 3: Table S5). Intriguingly, aspercryptins, trans-resorcylide, and azasperpyranone are exclusively present in the genome of *P. chrysogenum* 28R-6-F01 and absent in the tested and previous reported terrestrial strains [29, 32, 36, 37]. This suggests that *P. chrysogenum* may possess a wider array of secondary metabolic pathways, enabling it to gain a competitive advantage in resource-limited deep subseafloor environments.

In addition, comparative genome analysis revealed that strain 28R-6-F01 possesses eight unique genes and two expanded gene families related to secondary metabolism, distinguishing it from terrestrial strains (Additional file 2: Table S3, S4). Among these, four ABC transporter genes and three major facilitator superfamily (MFS) coding genes were identified, potentially contributing to penicillin synthesis and multi-drug resistance [34, 38, 39]. Two unique genes encoding versicolorin reductase were found, suggesting the strain’s capability to produce the toxic and carcinogenic mycotoxin aflatoxins [40, 41]. Furthermore, a novel gene annotated as the hepatitis delta virus antigen was discovered. As eukaryotic genomes contain numerous sequences of viral origin that have played diverse roles, such as facilitating horizontal gene transfer mediated by dsRNA viruses, conferring resistance to viruses, and contributing to the evolution of host organisms [42, 43], the discovery of the hepatitis delta virus antigen in the genome of *P. chrysogenum* suggests that this strain may possess certain antiviral capabilities.

#### DNA repair genes

Compared to terrestrial strains, strain 28R-6-F01 exhibits distinct genetic characteristics related to DNA repair processes. It possesses four unique genes and an expanded gene family associated with DNA repair, including Ankyrin repeat protein, SNF2 family protein, Hexokinase, NAD dependent epimerase/dehydratase, and UvrABC system protein families (Additional file 2: Table S3, S4). Ankyrin repeat proteins are involved in ubiquitylation signaling pathways (USP) that regulate various biological processes, such as DNA damage response and protein regulation [44]. SNF2 proteins play a crucial role in chromatin remodeling at DNA damage sites, which is essential for repairing DNA lesions [45]. Hexokinase is of particular importance for polysaccharides synthesis, which helps protect fungi from heat-induced damage [16]. NAD-dependent epimerase/dehydratase enzymes influence essential cellular functions, including DNA repair and chromatin remodeling [46]. UvrABC system protein A participates in nucleotide excision repair, which is vital for recognizing and repairing bulky DNA double-strand breaks [47]. Considering that strain 28R-6-F01 originates from deep subseafloor sediment environments, fungi inhabiting this environment face potential DNA damage caused by long-term exposure to high temperature, high pressure, and anaerobic stress [48–50]. Consequently, having a greater number and diversity of DNA repair genes may serve as an important mechanism for fungi to cope with the extreme environmental stress encountered in the deep subseafloor sedimentary environment.

#### Carbohydrate-active enzymes

Carbohydrate-active enzymes (CAZymes) are essential for fungi to break down various polysaccharides like cellulose, hemicellulose, pectin, and lignin, allowing them to obtain nutrients [51]. In the genome of *P. chrysogenum* 28R-6-F01, we identified a total of 385 CAZyme coding genes, including 206 glycoside hydrolases (GHs), 40 auxiliary activities (AAs), 75 glycosyl transferases (GTs), 17 carbohydrate esterases (CEs), 36 carbohydrate-binding modules (CBMs), and 11 polysaccharide lyase (PL) gene (Additional file 3: Table S6). Notably, three unique genes, including two GHs and one CBM, were absent in terrestrial strains (Additional file 2: Table S3).

Since subseafloor sediment environments have significantly less organic material compared to land surfaces, fungi that persist in these conditions may possess specialized carbohydrate metabolism capabilities to meet their survival and growth needs under such extreme circumstances [5, 41, 52, 53]. Therefore, the identification of specific hydrolase-encoding genes in *P. chrysogenum* 28R-6-F01 suggests that they may contribute to the fungus’s ability to survive for millions of years in subseafloor sedimentary environments where brown coal serves as the primary source of organic matter [5, 6].

### Structural comparison of *P. Chrysogenum* genomes

**Fig. 6:**
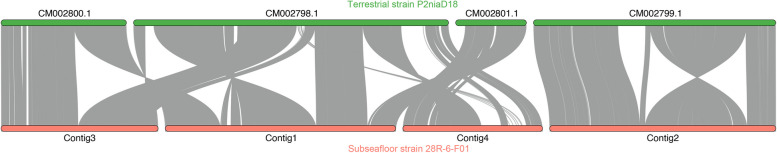
Genome collinearity analysis of *P. chrysogenum* strains 28R-6-F01 and P2niaD18. The green, and pink box represent the contigs of terrestrial strain P2niaD18, and subseafloor strain 28R-6-F01, respectively. Ligature represent different syntenic blocks

The genome structures of different *P. chrysogenum* strains were compared, showing high genomic similarity among nine strains except for P2niaD18 (Additional file 1: Fig. S5). A 4.42 Mb inversion on contig 2 of the 28R-6-F01 strain was observed, corresponding to the CM002799.1 region of the P2niaD18 strain (Fig. 6, Additional file 1: Fig. S6). KEGG enrichment analysis revealed that these inverted genes primarily participate in DNA repair, autophagy, and amino acid biosynthesis (Additional file 2: Table S5). These processes are associated with fungal adaptation to anaerobic environments [12, 13, 54, 55]. Hence, the enrichment of these genes in the 28R-6-F01 strain may contribute to its thriving in anaerobic deep subseafloor sedimentary environments.

### Phylogenetic analysis of *P. Chrysogenum* 28R-6-F01

**Fig. 7:**
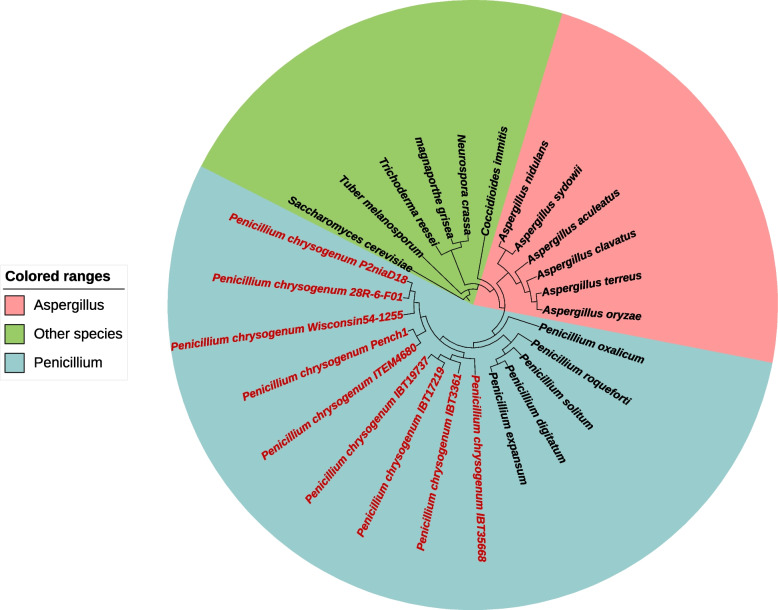
Phylogenetic tree of the *P. chrysogenum* and other sequenced filamentous fungi

A phylogenetic tree was constructed using 1129 conserved protein sequences from various *P. chrysogenum* strains. The tree revealed a distinct evolutionary branch encompassing all *Penicillium* species, with strains 28R-6-F01 and P2niaD18 clustering together (Fig. 7). Combining the tree analysis with fossil records, we estimated that the divergence between strain 28R-6-F01 and terrestrial strain P2niaD18 occurred approximately 35.4 million years ago (Additional file 1: Fig. S7). This timeframe aligns with the estimated age of the sedimentary environment (around 20 million years) where strain 28R-6-F01 was found [56].

## Conclusion

This study presents the first comprehensive de novo whole-genome sequencing and assembly of *P. chrysogenum* 28R-6-F01, which was isolated from coal-bearing sediment buried approximately 2.0 km beneath the seafloor. Comparative analysis with terrestrial *P. chrysogenum* strains revealed the presence of unique genes associated with secondary metabolite metabolism, DNA repair, and carbohydrate hydrolase, as well as the identification of three distinct secondary metabolism gene clusters. Additionally, significant expansions in gene families related to DNA repair were observed. These findings shed light on the adaptive selection mechanisms that have allowed fungi to thrive in the challenging coal-bearing subseafloor sediment for millions of years. The insights gained from this research contribute to our understanding of the lifestyle, evolution, and survival strategies of deep biosphere fungi.

## Methods

### Isolation and identification of 28R-6-F01

The fungal strain 28R-6-F01 utilized in this study was obtained from sediment samples collected at a depth of 2306 m below the seafloor from drilling Site C0020 (41°10.5983′N, 142°12.0328′E) near the Shimokita Peninsula, Japan [5]. The isolation and identification procedures for the fungus followed the methods described by Liu et al. [6]. Additional information about the sampling site is available in Additional file 3: Table S7.

### Assessment of fungal growth conditions

To investigate the growth of strain 28R-6-F01 under different culture conditions, single-factor experiments were conducted. The strain was cultured in MM medium at three temperatures (15, 30, and 45 °C), three pressures (0.1, 15, and 35 MPa), and three oxygen concentrations (0, 5, and 21%) for 1, 3, and 5 days to quantify the biomass [16]. The temperature and oxygen concentration experiments were carried out in shake flasks under 0.1 MPa, while the pressure experiment was conducted in a high pressure vessel (30 °C, 0% O_2_). Anaerobic conditions (0% O_2_) were generated by flushing the bottles with pure nitrogen gas (99.99%) for 10 min, followed immediately by sealing with a rubber stopper and aluminum cap. For 0.5, 5 and 21% O_2_, the bottles were flushed with a specific ratio of nitrogen and oxygen mixture [12]. The pure and mixed gases were purchased from Shangyuan Industrial Gas, China. Each treatment was replicated three times.

### Determination of antimicrobial activity

To assess the antimicrobial activity of *P. chrysogenum* 28R-6-F01, it was cultured in 250 mL flasks with 100 mL of PD medium (200 g/L potato and 20 g/L glucose) at 30 °C for 96 h with continuous shaking at 200 rpm. After cultivation, the culture supernatants were filtered using a 0.22 μm membrane (Millipore, USA) to remove hyphae. The resulting filtrate was then used for antibacterial and antifungal activity assays. For the antibacterial assay, small discs of filter paper soaked in the filtrate were placed on agar plates inoculated with *E. coli* (gram-negative) and *B. subtilis* (gram-positive) bacteria. The plates were incubated at 37 °C for 24 h to observe inhibition zones around the discs, indicating antibacterial activity [57]. Similarly, the antifungal activity was evaluated using the agar diffusion assay [34]. Wells were created in agar plates inoculated with *A. sydowii* and *P. capsici* fungi. The filtrate (60 μL) was added to the wells, and the plates were incubated at 30 °C for 72 h to observe inhibition of fungal growth around the wells. To ensure accuracy, positive controls using hygromycin and kanamycin, and blank controls using sterile water were included in the assays. Each treatment condition was replicated three times to account for experimental variations.

### Quantitative detection of penicillin

To assess the penicillin production capacity of strain 28R-6-F01, we cultured mycelia in a 300 mL medium containing the following components: pharmamedia 20 g/L, lactose 50 g/L, (NH_4_)_2_SO_4_ 4 g/L, CaCO_3_ 5 g/L, and phenylacetic acid 4 g/L [58]. Prior to sterilization, the pH was adjusted to 6.6. The culture was then incubated at 30 °C and 200 rpm for 3 days. After cultivation, the culture broth was centrifuged at 12,000 rpm for 10 min and then sterilized by filtering through a 0.22 μm membrane (Millipore, USA). A 1 mL volume of the filtrate was used to determine the quantity of penicillin produced by the strain using high-performance liquid chromatography (HPLC) [59]. The mobile phase buffer A (0.2 mol/L NaH_2_PO_4_, pH 3.5) and buffer B (100% methanol) with an isocratic method (40% of A). The HPLC system used was Shimadzu LC-20 HPLC (Shimadzu, Japan) equipped with an analytical 4.6 × 250 mm × 5 μm C18 column (Waters, USA).

### Genome sequencing and assembly

The mycelia used for DNA extraction were prepared by cultivating *P. chrysogenum* 28R-6-F01 in PD medium for 2 days at 30 °C and 200 rpm. The SDS method was used to extract genomic DNA from the mycelia of *P. chrysogenum* 28R-6-F01 [60]. A PacBio library with an insert size of 20 kb was constructed using the SMRT bell TM Template kit (Pacbio, USA), while the Illumina sequencing library for genome survey was generated using the NEBNext® UltraTM DNA Library Prep Kit (NEB, USA). The PacBio and Illumina libraries were sequenced using the PacBio Sequel and Illumina NovaSeq PE150 platform, respectively, at Beijing Novogene Bioinformatics Technology Co., Ltd. The Illumina raw reads were filtered for quality using FastQC v0.12.1 software with default parameters [61]. Based on k-mer statistics analysis, the clean reads were assembled for genome survey using SOAP denovo software [62]. The low-quality reads from PacBio sequencing were filtered using SMRT Link v5.0.1 [63], and the resulting clean reads were de novo assembled to obtain the original assembly contigs using the SMRT portal. The contigs were then optimized and upgraded using the variant Caller module of SMRT Link [63]. The quality of the genome assembly was evaluated by using Quast v5.0.2 software [64]. The mitochondrial sequence was assembled and annotated using Mitofinder v1.4.1 software [65], with the strain P2niaD18 mitochondrial genome (GeneBank: CM002802.1) as a reference genome.

### Gene prediction and annotation

For gene prediction, we utilized Augustus v3.3.3 and Genewise v2.4.1 software [66, 67]. Initially, Augustus was applied to the entire genomic sequence to generate an initial set of predicted genes. Then, Genewise was used on the gene prediction results of *P. chrysogenum* P2niaD18 to refine and supplement our predictions through homology searching [33]. The combined results were further processed using EVidenceModeler v2.10 and PASA v2.5.3 to obtain the final predicted genes [68, 69]. Repeat sequences were identified using RepeatMasker and Tandem repeats finder [70, 71]. tRNA and rRNA genes were predicted using tRNAscan-SE v2.0 and rRNAmmer v1.2, respectively [72, 73]. Additionally, sRNA, snRNA, and miRNA were predicted by performing BLAST v2.2.26 against the Rfam database [74]. Gene functions were annotated using NR, COG, SwissProt, GO, KEGG, and PFAM databases.

### Gene family identification and phylogenetic evolution analysis

A phylogenetic analysis was constructed using protein sequences from 19 species, including *Aspergillus aculeatus*, *A. clavatus*, *A. nidulans*, *A. oryzae*, *A. sydowii*, *A. terreus*, *P. chrysogenum*, *P. digitatum*, *P. expansum*, *P. oxalicum*, *P. roqueforti*, *P. rubens*, *P. solitum*, *Coccidioides immitis*, *Magnaporthe grisea*, *Neurospora crassa*, *S. cerevisiae*; *Trichoderma reesei*, and *Tuber melanosporum.* Multiple strains of *P. chrysogenum*, including 28R-6-F01, P2niaD18, Wisconsin 54–1255, IBT17219, IBT19737, IBT3361, IBT35668, ITEM4680, and Pench1 were also included in the analysis (Additional file 2: Table S6). OrthoFinder v2.4 software with the default parameters was used to classify protein families [75]. The protein sequences of all single-copy genes were aligned using MAFFT v7.407 and concatenated into a dataset [76]. Gblocks v0.91 was used for alignment optimization [77], and the resulting dataset was used to construct a phylogenetic tree using RAxML v8.2.12 [78]. Divergence times were estimated using MCMCTREE of PAML v4.9 package [79], and the divergence time between (i) *N. crassa* and *M. grisea* (146–219 mya) and (ii) *A. terreus* and *A. oryzae* (40.9–85.7 mya) from the TimeTree database was utilized for reference [80]. The phylogenetic tree and gene family clustering analyses were used to identify gene family expansions and contractions using CAFE [81]. The criteria defining significant expansion or contraction of gene families were a family-wide *p* < 0.05.

### KEGG enrichment

The OmicShare online tools, a free data analysis platform, was used to identify significantly overrepresented KEGG terms in this study. The significance level was set at a corrected *p* < 0.05.

### Genome collinearity analysis and genome information visualization

The collinearity analysis of *P. chrysogenum* 28R-6-F01 was conducted using the MUMmer v3.9.4 sequence alignment package [82]. The results were visualized using the TBtools v2.012 software and Dot online platform (https://dot.sandbox.bio/) [83]. Gene density of each chromosome was calculated using a sliding window file generated with BEDTools v2.25.0, with a window size of 100 kb [84]. The visualization of gene density, GC content, and duplication on the chromosomes was achieved using Circos v0.69 [85].

### Statistical analysis

Statistical analysis was performed by SPSS version 25.0 (SPSS, Chicago, IL, USA) and the significant threshold for all tests was set with *p* < 0.05 [16].

### Supplementary Information

**Additional file 1: Fig. S1. **Detection of penicillin by HPLC. **Fig. S2.** Standard curve of penicillin. **Fig. S3.** Comparative analysis of penicillin biosynthetic genes cluster in different *P. chrysogenum* strains. **Fig. S4.** Schematic representation of compartmentalization of penicillin biosynthetic pathway secretion of penicillin in *P. chrysogenum* 28R-6-F01. **Fig. S5.** Genome collinearity analysis of *P. chrysogenum* strain 28R-6-F01 and other strains. **Fig. S6.** Dot-plot of whole-genome alignment of *P. chrysogenum* strains 28R-6-F01 and P2niaD18. **Fig. S7.** Phylogenetic tree and divergence time of *P. chrysogenum* 28R-6-F01.

**Additional file 2: Table S1. **Comparison of the genomes of *P. chrysogenum* strain 28R-6-F01 and other strains. **Table S2. **Comparison of previous and new *P. chrysogenum* assemblies on genomic sequences level. **Table S3. **Annotation of specific genes in the 28R-6-F01 compared with other stains.** Table S4. **Functional annotation of expanded and contracted gene families in *P. chrysogenum* 28R-6-F01. **Table S5. **KEGG enrichment of inversion genes in the *P. chrysogenum* strain 28R-6-F01 compared with strain P2niaD18.** Table S6. **Resources of the 26 fungi for OrthoFinder analysis.

**Additional file 3: Table S1. **Statistical of predicted functional genes in public protein databases.** Table S2. **Summary statistics of repeat elements.** Table S3. **The number of all kinds of non-coding RNA.** Table S4. **The function classification of the secondary metabolic gene clusters.** Table S5. **Secondary metabolism gene clusters in *P. chrysogenum* 28R-6-F01.** Table S6. **Statistical of predicted functional genes in Cazymes databases. **Table S7.** The sampling site information.

## Data Availability

Raw Pacbio reads for the genome assembly have been deposited under BioProject accession number PRJNA983921, Illumina raw data were given accession PRJNA983944 for the genome size survey. Genome assembly of *P. chrysogenum* 28R-6-F01 is deposited in the NCBI genome database under the accession PRJNA984597. The sequences of *P. chrysogenum* 28R-6-F01 mitochondrial genome and 17 genes have been upload into github database (https://github.com/liuxuan-425lab/P.chrysogenum-28R-6-F01).

## Abbreviations

BLAST: Basic Local Alignment Search Tool
bp: base pairs
BUSCO: Benchmarking Universal Single-Copy Orthologs
LTR: Long Terminal Repeat
Mb: Mega Base Pairs
Mya: Million years ago
NCBI: National Center for Biotechnology Information
TE: Transposable Element
GATK: Genome Analysis Toolkit
SNP: Single Nucleotide Polymorphism
Quast: Quality Assessment Tool for Genome Assemblies
TR: Tandem Repeats
PDA: Potato dextrose Agar
PD: PDA without agar
GO: Gene Ontology
KEGG: Kyoto Encyclopedia of Genes and Genomes
COG: Eukaryotic Clusters of Orthologous Groups
SwissProt: SWISS-PROT Protein Knowledgebase
PFAM: Protein families database
