# Supplementary material for: Genomic insights into Penicillium chrysogenum adaptation to subseafloor sedimentary environments
Source: BMC Genomics. 2024 Jan 2;25:4. doi: 10.1186/s12864-023-09921-1 (PMC10759354; doi:10.1186/s12864-023-09921-1)
Supplement: Supplementary file 1 — Additional file 1: Fig. S1. Detection of penicillin by HPLC. Fig. S2. Standard curve of penicillin. Fig. S3. Comparative analysis of penicillin biosynthetic genes cluster in different P. chrysogenum strains. Fig. S4. Schematic representation of compartmentalization of penicillin biosynthetic pathway secretion of penicillin in P. chrysogenum 28R-6-F01. Fig. S5. Genome collinearity analysis of P. chrysogenum strain 28R-6-F01 and other strains. Fig. S6. Dot-plot of whole-genome alignment of P. chrysogenum strains 28R-6-F01 and P2niaD18. Fig. S7. Phylogenetic tree and divergence time of P. chrysogenum 28R-6-F01. [file 12864_2023_9921_MOESM1_ESM.docx]

**Supplementary Materials**

**Genomic insights into *Penicillium chrysogenum* adaptation to subseafloor sedimentary environments**

Xuan Liu^1^, Xinran Wang^1^, Fan Zhou^1^, Yarong Xue^1^, Changhong Liu^1*^

^1^State Key Laboratory of Pharmaceutical Biotechnology, Nanjing University, Nanjing 210023, China

^*^Corresponding author

**Supplemental Figures**


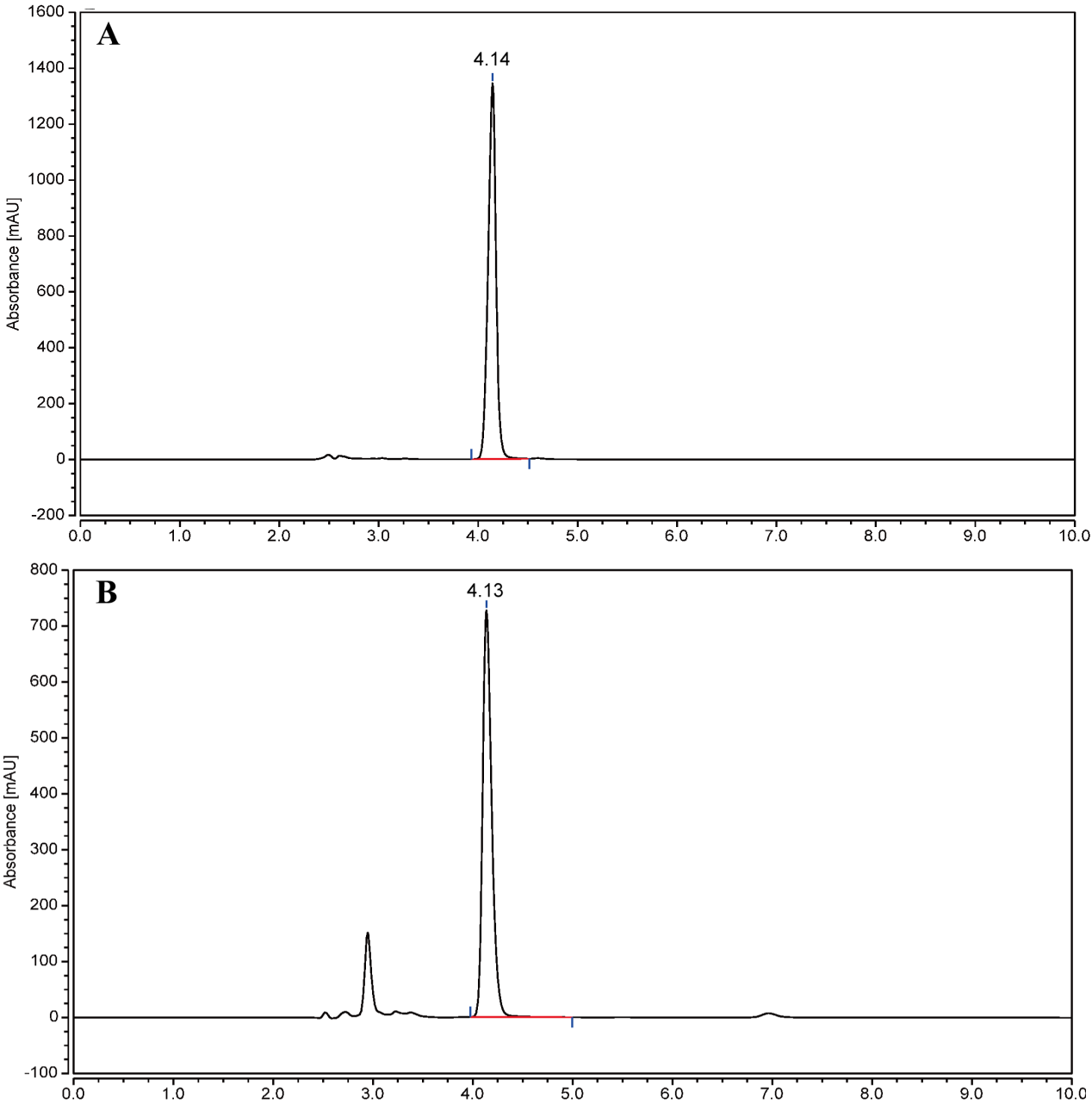


**Fig. S1. Detection of penicillin by HPLC.** The formation of penicillin by *P. chrysogenum* 28R-6-F01 (B) and positive control (A).


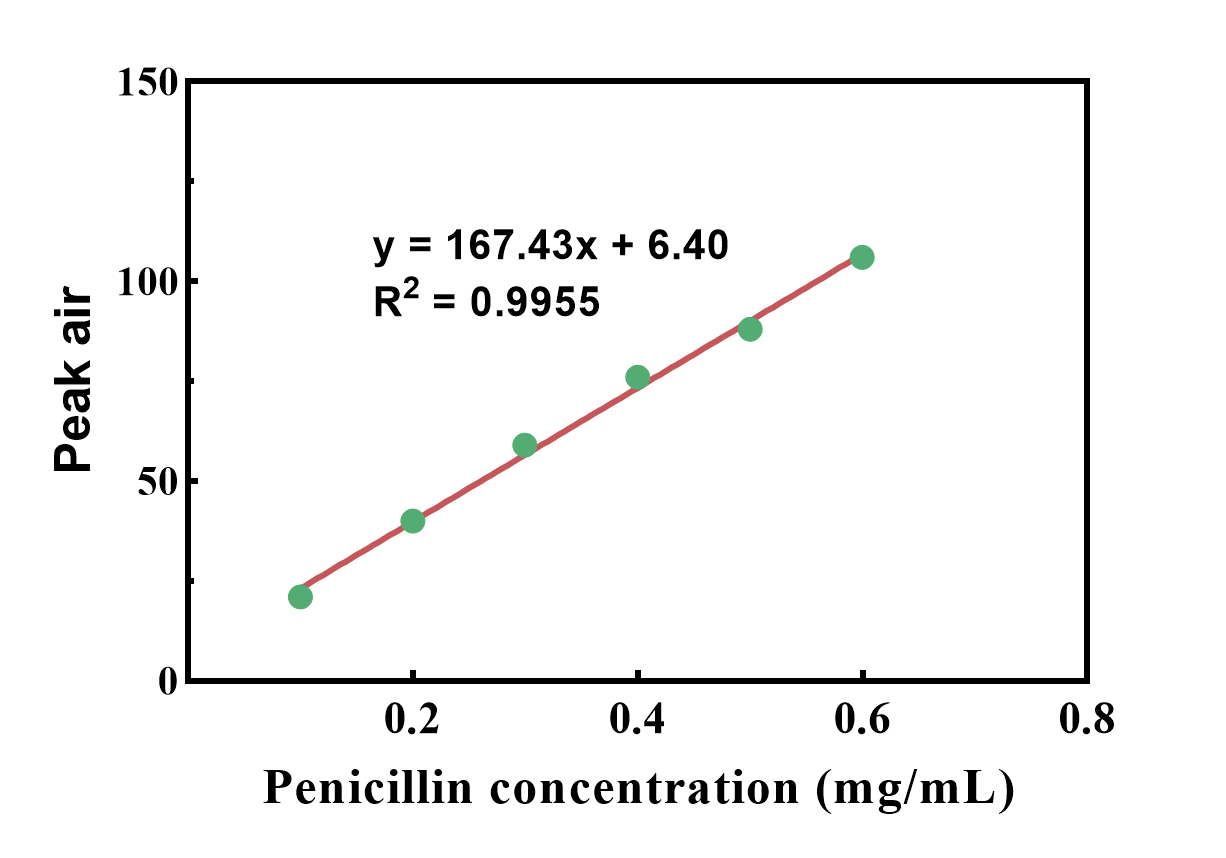


**Fig. S2. Standard curve of penicillin.**


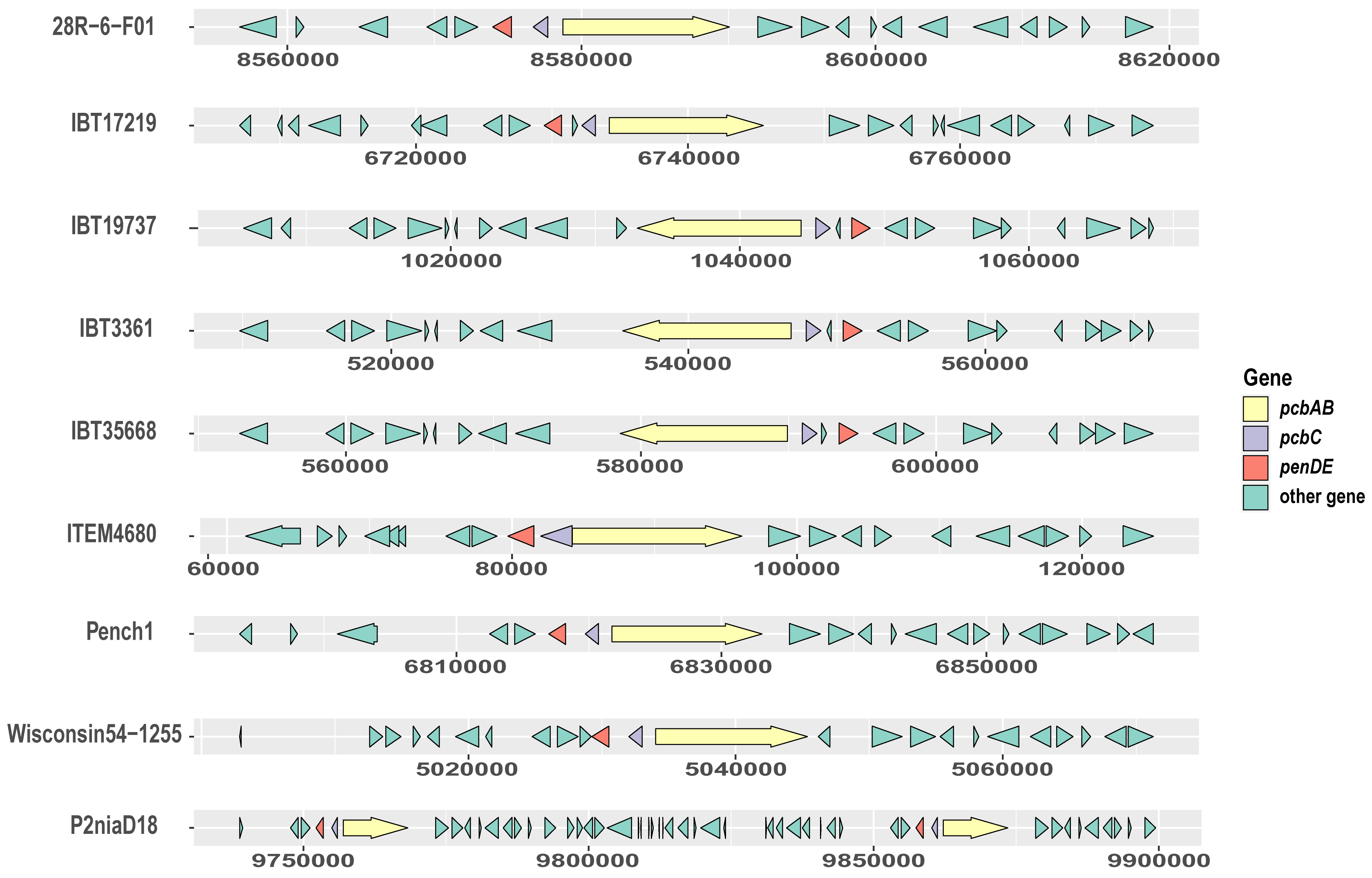


**Fig. S3. Comparative analysis of penicillin biosynthetic genes cluster in different *P. chrysogenum* strains.**


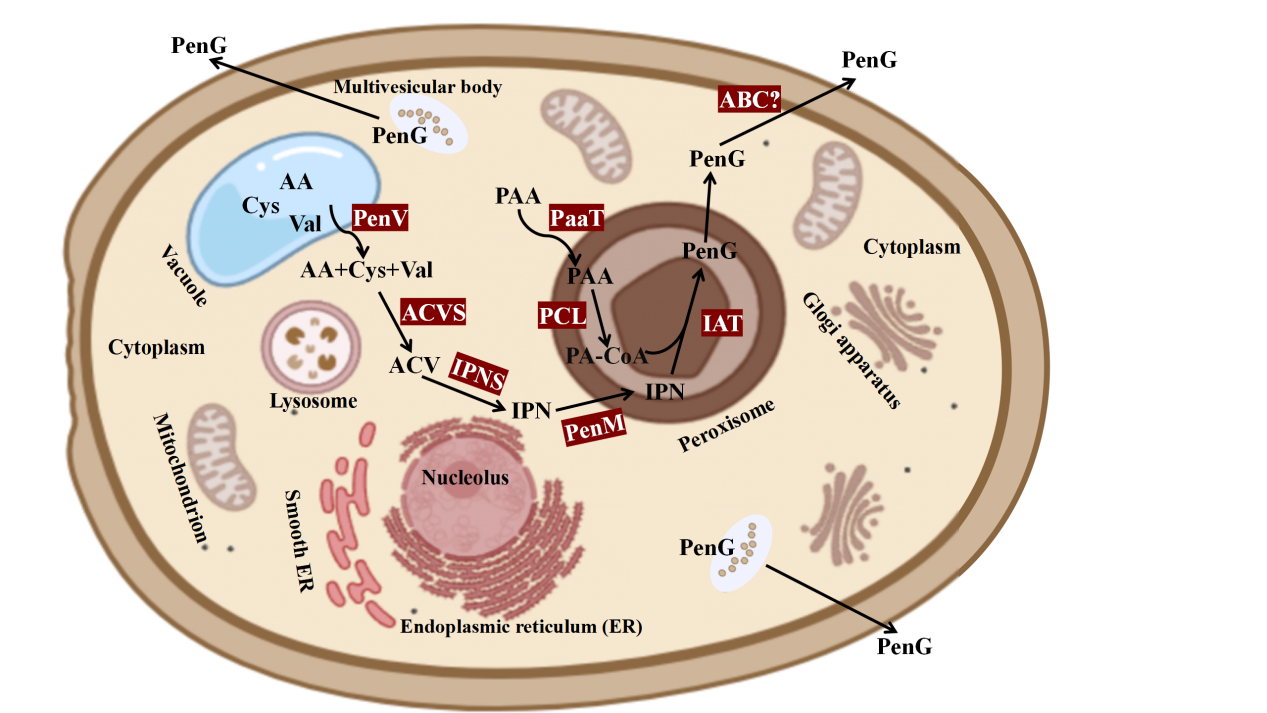


**Fig. S4. Schematic representation of compartmentalization of penicillin biosynthetic pathway secretion of penicillin in *P. chrysogenum* 28R-6-F01.**

Cys: L-cystine, AAA: a-aminoadipic acid, Val: L-valine, PAA: Phenylacetic acid, ACVS: δ-(L-α-Aminoadipyl)-L-cysteinyl-D-valine, IPN: Isopenicillin N, PenG: Penicillin G, ACVS: δ-(L-α-Aminoadipyl)-L-cysteinyl-D-valine synthetase, IPNS: Isopenicillin N synthase, PCL: phenylacetyl CoA ligase, IAT:Isopenicillin N CoA acyl transferase.


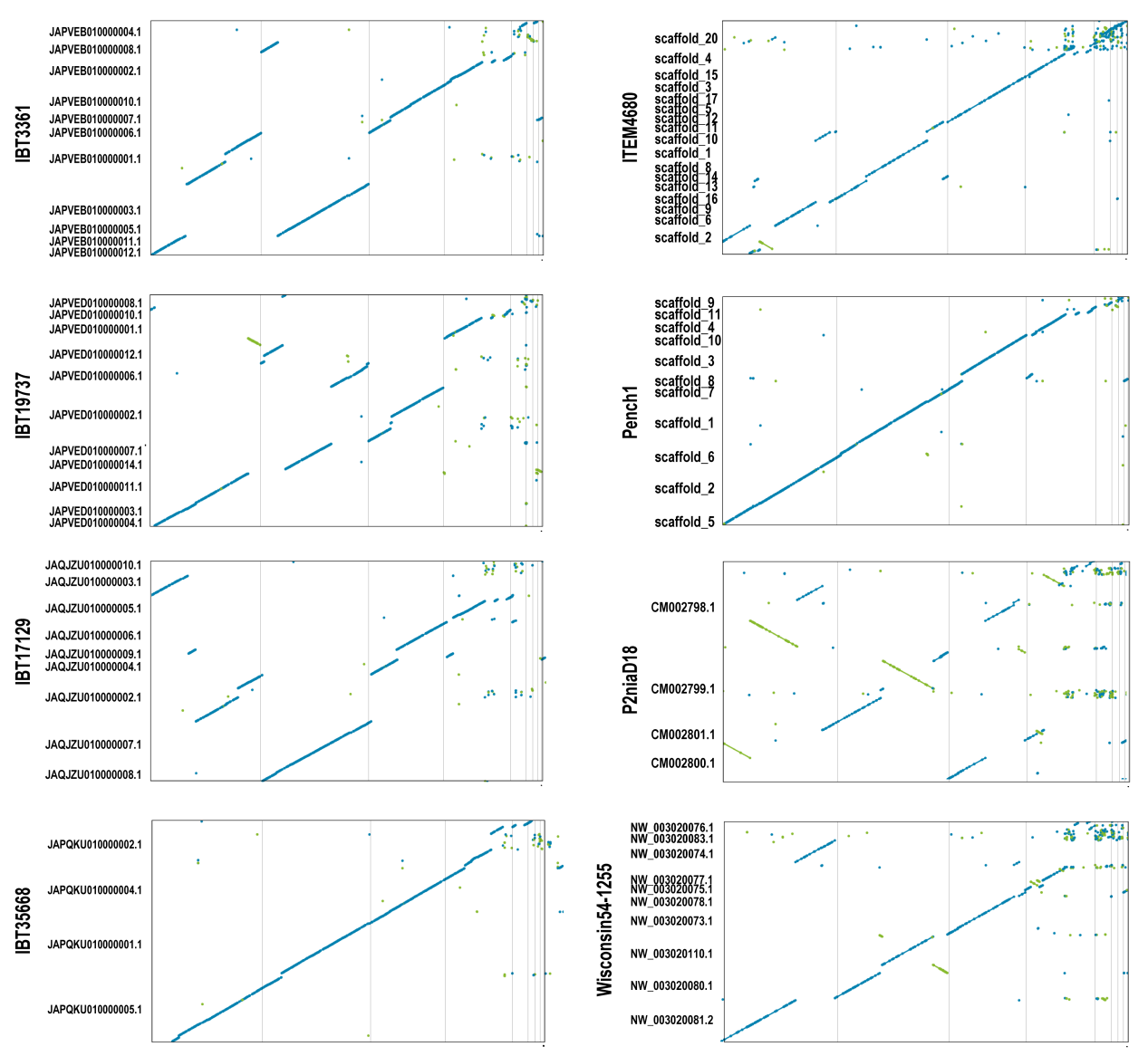


**Fig. S5. Genome collinearity analysis of *P. chrysogenum* strain 28R-6-F01 and other strains.** Blue dots represent forward alignments, whereas green dots represent reverse alignments.


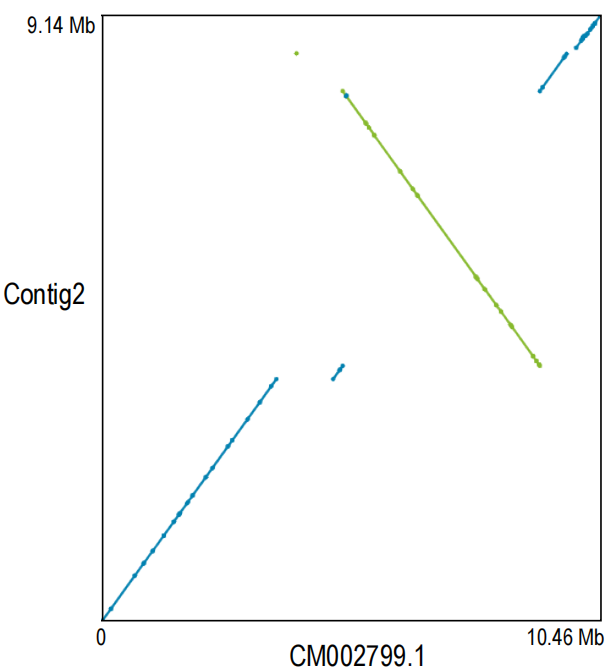


**Fig. S6. Dot-plot of whole-genome alignment of *P. chrysogenum* strains 28R-6-F01 and P2niaD18.** Blue dots represent forward alignments, whereas green dots represent reverse alignments.


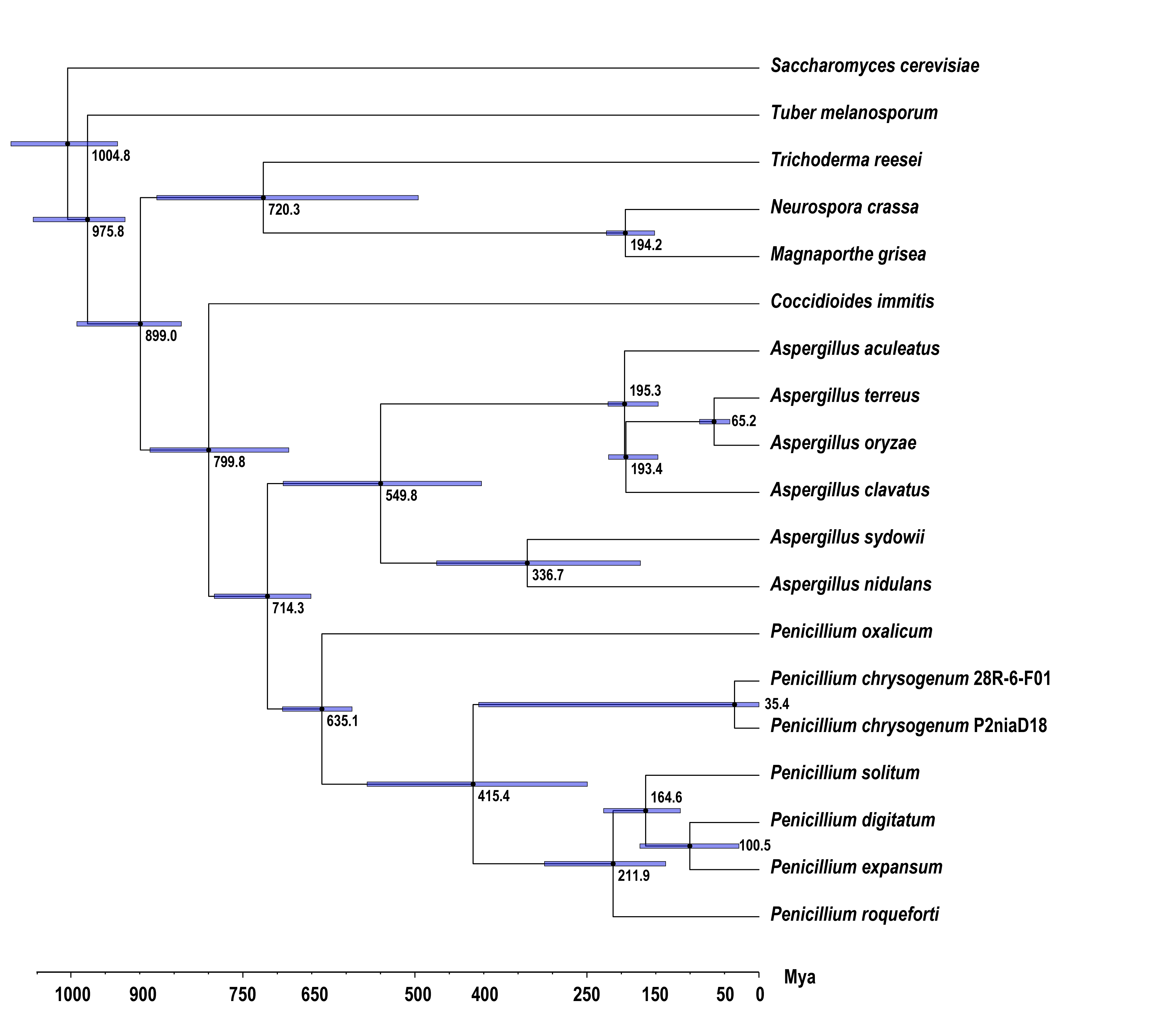


**Fig. S7. Phylogenetic tree and divergence time of *P. chrysogenum* 28R-6-F01.** The branch lengths of the phylogenetic tree are scaled to estimated divergence time. The blue bars on the nodes indicate the 95% credibility intervals of the estimated posterior distributions of the divergence times. The overall timeline is shown below the phylogenetic tree.
