# Supplementary material for: Genomic insights into Penicillium chrysogenum adaptation to subseafloor sedimentary environments
Source: BMC Genomics. 2024 Jan 2;25:4. doi: 10.1186/s12864-023-09921-1 (PMC10759354; doi:10.1186/s12864-023-09921-1)
Supplement: Supplementary file 3 — Additional file 3: Table S1. Statistical of predicted functional genes in public protein databases. Table S2. Summary statistics of repeat elements. Table S3. The number of all kinds of non-coding RNA. Table S4. The function classification of the secondary metabolic gene clusters. Table S5. Secondary metabolism gene clusters in P. chrysogenum 28R-6-F01. Table S6. Statistical of predicted functional genes in Cazymes databases. Table S7. The sampling site information. [file 12864_2023_9921_MOESM3_ESM.docx]

**Supplementary Materials**

**Genomic insights into *Penicillium chrysogenum* adaptation to subseafloor sedimentary environments**

Xuan Liu^1^, Xinran Wang^1^, Fan Zhou^1^, Yarong Xue^1^, Changhong Liu^1*^

^1^State Key Laboratory of Pharmaceutical Biotechnology, Nanjing University, Nanjing 210023, China

^*^Corresponding author

**Supplemental Tables**

**Table S1. Statistical of predicted functional genes in public protein databases.**

| **NR** | **SwissProt** | **KEGG** | **COG** | **GO** | **Pfam** |
| --- | --- | --- | --- | --- | --- |
| 6,645 | 3,023 | 6,604 | 2,046 | 4,759 | 4,759 |

**Table S2. Summary statistics of repeat elements.**

| **Type** | **Number** | **Total Length(bp)** | **In Genome(%)** | **Average length(bp)** |
| --- | --- | --- | --- | --- |
| LTR | 1,291 | 208,804 | 0.6291 | 166 |
| DNA | 687 | 198,432 | 0.5978 | 293 |
| LINE | 460 | 223,290 | 0.6727 | 489 |
| SINE | 100 | 5,904 | 0.0178 | 61 |
| RC | 31 | 1,985 | 0.006 | 64 |
| Unknown | 8 | 875 | 0.0026 | 109 |
| Total | 2,577 | 634,402 | 1.9113 | 252 |

**Table S3. The number of all kinds of non-coding RNA.**

| **Type** | **Number** | **Average length(bp)** | **Total length(bp)** |
| --- | --- | --- | --- |
| tRNA | 186 | 87 | 16,210 |
| 5s | 42 | 116 | 4,872 |
| 5.8s | 0 | 0 | 0 |
| 18s | 0 | 0 | 0 |
| 28s | 0 | 0 | 0 |
| sRNA | 3 | 211 | 633 |
| snRNA | 34 | 137 | 4,666 |
| miRNA | 0 | 0 | 0 |

**Table S4. The function classification of the secondary metabolic gene clusters.**

| **Secondary_Metabolism** | **NRPS** | **NRPS-like** | **T1PKS** | **terpene** | **indole** | **betalactone** | **NRP-metallophore** | **Total** |
| --- | --- | --- | --- | --- | --- | --- | --- | --- |
| 28R-6-F01 | 12 | 11 | 16 | 3 | 1 | 1 | 1 | 45 |
| IBT3361 | 12 | 13 | 12 | 3 | 1 | 2 | 1 | 44 |
| IBT19737 | 12 | 12 | 15 | 3 | 1 | 2 | 1 | 46 |
| IBT35668 | 12 | 12 | 15 | 2 | 1 | 2 | 1 | 45 |
| IBT17219 | 13 | 11 | 16 | 3 | 1 | 2 | 0 | 46 |
| Pench1 | 11 | 15 | 15 | 5 | 1 | 2 | 1 | 50 |
| ITEM4680 | 12 | 11 | 17 | 3 | 1 | 1 | 1 | 46 |
| P2niaD18 | 13 | 10 | 17 | 3 | 1 | 2 | 1 | 47 |
| Wisconsin54-1255 | 11 | 11 | 15 | 3 | 1 | 2 | 1 | 44 |

**Table S5. Secondary metabolism gene clusters in *P. chrysogenum* 28R-6-F01.**

| **Type** | **From** | **To** | **Most similar known cluster** |
| --- | --- | --- | --- |
| NRPS-like | 1466341 | 1519957 |  |
| T1PKS | 1902698 | 1964656 | patulin |
| NRPS-like | 2267339 | 2311073 |  |
| NRPS | 4819826 | 4876806 | metachelin C/metachelin A/metachelin A-CE/metachelin B/dimerumic acid 11-mannoside/dimerumic acid |
| T1PKS | 5614169 | 5670943 |  |
| terpene | 5720831 | 5752482 |  |
| T1PKS | 6417842 | 6485784 |  |
| NRP-metallophore,NRPS | 6642647 | 6720955 |  |
| T1PKS | 7444560 | 7512968 |  |
| NRPS | 8890545 | 8965356 |  |
| NRPS-like | 695158 | 754256 |  |
| NRPS-like | 3039236 | 3093100 |  |
| NRPS-like | 3110324 | 3170747 |  |
| T1PKS | 3725432 | 3791696 |  |
| T1PKS | 4496816 | 4558391 | YWA1 |
| NRPS,indole | 4621812 | 4677746 | histidyltryptophanyldiketopiperazine/dehydrohistidyltryptophanyldiketopiperazine/roquefortine D/roquefortine C/glandicoline A/glandicoline B/meleagrine |
| T1PKS | 4710121 | 4770676 |  |
| terpene | 4823741 | 4852621 |  |
| terpene | 4988005 | 5019484 | squalestatin S1 |
| NRPS,T1PKS | 5274046 | 5387518 | trans-resorcylide |
| NRPS | 5717354 | 5793436 | aspercryptins |
| T1PKS | 7065909 | 7194735 | sorbicillin |
| T1PKS | 7324062 | 7412235 |  |
| NRPS | 7864425 | 7920383 |  |
| NRPS | 8548880 | 8620109 | δ-(L-α-aminoadipyl)-L-cysteine-D-valine/isopenicillin N/benzylpenicillin/phenoxymethylpenicillin |
| NRPS-like | 8812230 | 8905670 |  |
| betalactone | 8982955 | 9023490 |  |
| T1PKS | 1220539 | 1287559 |  |
| NRPS-like | 2250136 | 2308855 |  |
| NRPS-like | 3962688 | 4023414 | choline |
| NRPS | 6282554 | 6342058 |  |
| T1PKS,NRPS | 853706 | 984805 | nidulanin A |
| T1PKS,NRPS | 1184001 | 1258660 | metachelin C/metachelin A/metachelin A-CE/metachelin B/dimerumic acid 11-mannoside/dimerumic acid |
| NRPS-like | 1887375 | 1946275 | azasperpyranone A/azasperpyranone B/azasperpyranone C/azasperpyranone D/azasperpyranone E/azasperpyranone F/azasperpyranone G/azasperpyranone H |
| NRPS-like | 2010049 | 2070960 |  |
| T1PKS | 2219636 | 2281592 | chrysoxanthone A/chrysoxanthone B/chrysoxanthone C |
| T1PKS,NRPS | 2887517 | 2959650 |  |
| NRPS-like | 4090006 | 4153083 | FR901483 |
| T1PKS | 55080 | 120582 | 4-oxomacrophorin A/macrophorin A/5'-epimacrophorin B |

**Table S6. Statistical of predicted functional genes in Cazymes databases.**

| **CAZymes_class** | **Match_number** |
| --- | --- |
| CBM | 36 |
| CE | 17 |
| GH | 206 |
| GT | 75 |
| PL | 11 |
| AA | 40 |

**Table S7. The sampling site information**

| **Sampling site information** | ***Penicillium chrysogenum* 28R-6-F01** |
| --- | --- |
| Lithology | sand |
| Depth | 2,306 |
| Porosity | 0.2831 |
| Temperature | 49 |
| PH | 7.88 |
| H_2_ | 24.3 |
| CO | 5740 |
| TN | 0.1 |
| TC | 2.9 |
| TOC | 2.3 |
| TOC-TN | 38 |
| Inorganic carbon | 0.6 |
| Methane | 39.4 |
| Ethane | 4.8 |
| CaCO_3_ | 5.1 |
